# Supplementary material for: Check the Need–Prevalence and Outcome after Transvenous Cardiac Implantable Electric Device Extraction without Reimplantation
Source: J Clin Med. 2021 Sep 7;10(18):4043. doi: 10.3390/jcm10184043 (PMC8467543; doi:10.3390/jcm10184043)
Supplement: Supplementary file 1 [file jcm-10-04043-s001.zip › jcm-1350643-supplementary.pdf]

Supplemental material

Supplemental Table S1. Procedural outcome.

	Total population (n=223)	Reimplantation (n=190)	Non-reimplantation (n=33)	p-value
Extraction tools				0.223
Manual traction	64.1% (n=143)	65.8% (n=125)	54.6% (n=18)	
Mechanical sheath	34.1% (n=76)	32.1% (n=61)	45.5% (n=15)	
Laser sheath	1.8% (n=4)	2.1% (n=4)	0% (n=0)	
Complete removal, n (%)	95.5% (n=213)	94.7% (n=180)	100% (n=33)	0.365
Clinical success, n (%)	99.6% (n=222)	100.0% (n=190)	97.0% (n=32)	0.148
Major complications	3.1% (n=7)	2.6% (n=5)	6.1% (n=2)	0.277
Minor complications	3.1% (n=7)	2.6% (n=5)	6.1% (n=2)	0.277
Blood transfusion	4.5% (n=10)	3.2% (n=6)	12.1% (n=4)	0.044*
Days until discharge	8 (3-23)	7 (3-21)	14 (5-26)	0.204

Supplemental Table S2. Individual patient characteristics of patients without reimplantation.

No	Age, gender	Device	Implantation indication	Device/lead age (months)	Indication for explant	Reason for not-explant	Follow up
1	56, M	ICD	DCM	16 / 117	endocarditis	restoration of LV function	alive after 3 months
2	25, M	ICD	ARVD	68 / 19	pocket infection	negative electrophysiological study and MRI	alive after 13 months
3	19, M	ICD	SCD	11 / 11	pocket infection	negative electrophysiological study and MRI	received ILR alive after 110 months
4	65, M	CRT-D	DCM	93 / 93	pocket infection	persistent infection	alive after 6 months
5	57, M	CRT-D	ICM	92 / 92	pocket infection	restoration of LV function	alive after 18 days
6	75, M	ICD	DCM	16 / 16	pocket infection	negative electrophysiological study, restoration of LV function	alive after 45 months

7	94, M	PM	SSS	78 / 78	endocarditis	no pauses at ECG monitoring	**death after 43 months (senile decay)**
8	80, M	PM	SSS	67 / 67	pocket infection	no pauses at ECG monitoring	alive after 118 months
9	40, M	ICD	DCM	104 / 104	non functional lead	negative electrophysiological study	received ILR **reimplant after 17 months (recurrence of arrhythmia)** alive after 117 months
10	68, M	CRT-D	DCM	78 / 78	pocket infection	persistent infection	alive after 85 months
11	73, M	CRT-D	ICM	43 / 43	endocarditis	persistent infection	alive after 16 months
12	74, M	ICD	DCM	105 / 105	endocarditis	persistent infection	alive after 34 months

13	74, M	CRT-D	DCM	82 / 82	pocket infection	patient preference	**death after 39 months (Parkinson's disease)**
14	65, F	PM	SSS	86 / 86	lead interference	no pauses at ECG monitoring	alive after 5 days
15	43, F	ICD	Fallot Tetralogy	170 / 170	non functional lead	restoration of LV function, pulmonary valve replacement	**reimplant after 108 months (recurrent VTs)**<br>alive after 108 months
16	80, M	PM	SSS	243 / 243	pocket infection	no pauses at ECG monitoring	**death after 33 months (advanced heart failure)**
17	62, M	ICD	ICM	52 / 52	endocarditis	patient preference	**death after 34 months (cancer)**

18	44, M	ICD	Brugada	78 / 78	endocarditis	persistent infection	alive after 105 months
19	80, M	PM	SSS	7 / 7	pocket infection	persistent infection	alive after 101 months
20	54, F	CRT-D	DCM	93 / 93	pocket infection	restoration of LV function	alive after 91 months
21	63, M	ICD	ICM	105 / 45	pocket infection	negative electrophysiological study	alive after 16 months
22	82, M	PM	syncope	7 / 7	pain from implanted PM	patient preference	received ILR alive after 14 months
23	76, M	ICD	ICM	22 / 22	endocarditis	persistent infection	alive after 3 months
24	33, M	ICD	VT idiopathic	80 / 80	non functional lead	negative electrophysiological study	alive after 12 months

25	74, M	CRT-D	DCM	138 / 150	pocket infection	restoration of LV function	received ILR<br>**reimplant after 22 months (deterioration of LV function)**<br>alive after 23 months
26	78, M	PM	SSS	58 / 58	non functional lead	no pauses at ECG monitoring	alive after 80 months
27	79, F	CRT-D	DCM	0 / 0	pocket infection	persistent infection, patient preference	alive after 3 months
28	82, F	PM	SSS	81 / 81	pocket infection	persistent infection	alive after 2 months
29	79, M	PM	SSS	24 / 216	pocket infection	no pauses at ECG monitoring	received ILR<br>alive after 74 months
30	76, M	CRT-D	DCM	20 / 144	endocarditis	persistent infection, restoration of LV function	alive after 68 months
31	72, M	CRT-D	DCM	2 / 72	endocarditis	negative electrophysiological study	alive after 6 months

32	73, M	PM	SSS	12 / 60	endocarditis	no pauses at ECG monitoring	alive after 69 months
33	68, M	CRT-D	DCM	36 / 108	endocarditis	persistent infection	**reimplant after 56 months (worsening symptoms)** alive after 66 months
